# A CT-Based scoring system for predicting degenerative posterosuperior rotator cuff tears: a risk stratification tool for patients with contraindications to MRI

**DOI:** 10.1186/s12891-026-09874-y

**Published:** 2026-04-20

**Authors:** Xieyu Wang, Guihu Liu, Xiaolong Wang, Haibin Zhou, Guangsi Shen

**Affiliations:** 1 Department of Orthopedics, The Second Affiliated Hospital of Soochow University, Suzhou, Jiangsu People’s Republic of China; 2 Department of Orthopedics, Soochow New District People’s Hospital, Suzhou, Jiangsu People’s Republic of China

**Keywords:** Rotator Cuff Tear, Computed Tomography, Critical Shoulder Angle, Fatty Infiltration, Acromial Index, Risk Score

## Abstract

**Background:**

Rotator cuff tears (RCT) are a primary cause of shoulder pain and a leading source of shoulder disability in later stages. Magnetic resonance imaging (MRI) is the diagnostic gold standard but is not always feasible. Computed tomography (CT) is often available but provides limited direct soft-tissue information. Although various CT-derived measurements of the shoulder have been identified as predictors for RCT, we hypothesize that a combination of predictors will provide superior diagnostic and predictive performance compared to individual predictors. The primary objective of this study was to develop and validate a scoring system based on these factors to estimate the likelihood of a degenerative posterosuperior rotator cuff tear in patients for whom MRI is unavailable or contraindicated.

**Methods:**

This retrospective study analyzed 326 cases who underwent both shoulder CT and MRI examinations at our hospital. MRI was the reference standard, dividing patients into a tear group (full-thickness tear of supraspinatus/infraspinatus) and a control group (intact cuff). Candidate predictors included: age, sex, Body mass index (BMI), symptom duration, physical exam findings (Hug-up test, Drop arm sign, Jobe test, External rotation lag test), and CT-derived parameters including the Critical shoulder angle (CSA), Acromial index (AI), Goutallier grade of fatty infiltration, Supraspinatus occupation ratio, and the Hounsfield unit (HU) ratio of the deltoid to supraspinatus muscle, sex, age, Duration of symptoms(DOS), BMI and Physical examination findings(Hug-up test, Drop arm sign, Jobe test, External rotation lag test and Hawkins test). These factors were analyzed using univariate and multivariate analyses. A weighted scoring system was developed based on the odds ratios (OR) from the multivariate model. Model performance was assessed using the area under the receiver operating characteristic curve (AUC).

**Results:**

Multivariate analysis identified eight independent risk factors of posterosuperior rotator cuff tear (RCT-PT): age (*p* < 0.01, OR = 1.090), symptom duration (*p* = 0.012, OR = 1.036), fatty infiltration grade (*p* = 0.047, OR = 2.252), critical shoulder angle (*p* = 0.028, OR = 1.175), acromial index (*p* = 0.034, OR = 1.068), supraspinatus occupation ratio (*p* < 0.01, OR = 0.880), and physical examination findings including the Hug-up test (*p* < 0.01, OR = 11.061), Drop arm sign (*p* = 0.036, OR = 3.124), and External Lag test (*p* < 0.01, OR = 4.558). Subsequently, a 12-point scoring system was developed. The score demonstrated excellent discriminatory ability with an AUC of 0.930. A cut-off score of 6.5 yielded a sensitivity of 86.1% and specificity of 86.4% for predicting RCT-PT.

**Conclusion:**

This CT-based scoring system, integrating morphological parameters with clinical factors, provides a useful tool for risk stratification of posterosuperior rotator cuff tears. It offers a complementary decision-support aid for clinicians when MRI is not possible, such as in pre-operative planning for shoulder arthroplasty, helping to identify patients who may warrant more definitive investigation or influence surgical strategy.

## Background

A rotator cuff tear is a common pathological condition that causes shoulder pain and functional impairment, and can even lead to disability in severe cases [1, 2]. Accurate diagnosis of rotator cuff tears is of particular importance. Magnetic resonance imaging is considered the preferred imaging modality for evaluating the rotator cuff, offering comprehensive assessment of both tendinous and muscular structures [3]. Shoulder radiographs can serve as an initial tool to assess bony abnormalities associated with shoulder impingement. However, MRI is contraindicated in some patients (e.g., those with certain pacemakers, implants, or severe claustrophobia) and may not be readily available or cost-effective in all clinical settings. In such scenarios, alternatives like ultrasound (US) or CT arthrography offer high diagnostic accuracy for full-thickness tears. Computed tomography, on the other hand, is excellent for evaluating bony details and detecting the presence of gas and calcific deposits [3].

For instance, such cases may include patients with cardiac pacemakers, implantable drug infusion pumps, magnetic aneurysm or vascular clips, claustrophobia, or critically ill patients in the ICU. Given that a CT scan may be the only advanced imaging available, particularly in patients being evaluated for shoulder arthroplasty, there is a clinical need to maximize the diagnostic information it can provide about the rotator cuff. In these scenarios, shoulder joint CT represents the optimal alternative, as various parameters derived from the imaging can effectively predict the risk of rotator cuff tears, thereby allowing preliminary assessment of the rotator cuff condition. Numerous studies have confirmed the correlation of the Critical Shoulder Angle (CSA) and Acromial Index (AI) with rotator cuff tears, establishing them as reliable predictive factors [4–8]. Supporting this, Simone et al. reported a mean CSA of 36.7° in rotator cuff tear patients, with a clear trend of increasing tear severity alongside greater CSA values [4].

Chronic rotator cuff tears are consistently associated with muscle atrophy and fatty infiltration [9–11]. Worsening fatty infiltration and muscle atrophy are closely linked to the severity of rotator cuff tears, making them critical indicators for predicting tear extent, assessing the feasibility of surgical repair, and determining patient outcomes [12–14]. While current studies indicate a link between sarcopenia and rotator cuff tears [15]. the causal relationship remains unclear. Furthermore, the relationship between systemic muscle health, reflected in the deltoid muscle, and tear-specific changes in the supraspinatus is complex [15, 16]. Reem et al. further demonstrated that rotator cuff tear-induced muscle atrophy disproportionately affects the deltoid and supraspinatus muscles. Consequently, using the deltoid as a reference for comparative studies may yield more reliable results than studying the supraspinatus in isolation [16].

Therefore, we aim to determine whether the Hounsfield Unit (HU) ratio of the deltoid to supraspinatus muscle can serve as a predictor for rotator cuff tears and to develop a systematic scoring system based on shoulder CT for assessing the risk of rotator cuff tears.

## Methods

### Patient selection

This study was a retrospective cohort analysis of consecutive patients who underwent both shoulder CT and MRI at the authors’ institution between January 2021 and January 2024(the interval between CT and MRI examinations was no more than one month). The study protocol was approved by the Institutional Review Board. MRI findings served as the reference standard. The “tear” group comprised patients with a full-thickness tear of the supraspinatus and/or infraspinatus tendons (posterosuperior tear) and an intact subscapularis tendon. The “control” group comprised patients with an intact rotator cuff on MRI. The inclusion criteria were patients with: (1) an MRI-confirmed posterosuperior rotator cuff tear. (2) availability of both shoulder CT and MRI examinations. (3) an intact subscapularis tendon. Patients were excluded based on the following criteria: (1) a history of trauma. (2) an isolated subscapularis tendon tear or any tear involving the subscapularis. (3) a history of shoulder fracture or tumor. (4) an irreparable rotator cuff tear (defined on MRI by Goutallier grade ≥ 3, a positive tangent sign, or massive retraction to the glenoid rim), and (5) incomplete baseline data.

### Clinical variables

We documented patient variables including age, sex, body mass index, duration of symptoms and examination findings (such as the Hug-up test, Drop arm sign, Jobe test, External rotation lag test and Hawkins test). Systemic conditions such as diabetes, hypertension, and frozen shoulder were also recorded.

### Image acquisition

MRI examinations were performed on a 3.0-T scanner (MAGNETOM Skyra, Siemens Healthcare) using a dedicated shoulder coil. The protocol included proton-density weighted fat-suppressed sequences in the oblique coronal, oblique sagittal, and axial planes. CT examinations were performed on a 128-slice scanner (SOMATOM Definition Flash, Siemens Healthcare). Images were acquired with the patient supine and the arm in a neutral position, using a standard bone and soft tissue algorithm with slice thickness of 0.6 mm reconstructed at 1.0 mm intervals in axial, sagittal, and coronal planes.

### CT image analysis and measurement of predictors

All CT measurements were performed by a single orthopedic surgeon (X.W.) with 5 years of experience in shoulder imaging, who was blinded to the MRI findings and all clinical data. To ensure standardization, all measurements were performed on a PACS workstation (Carestream Health). For each parameter, measurements were performed three times, and the average was used for analysis. A second observer (G.L.) repeated all measurements on a randomly selected subset of 30 patients to assess interobserver reliability. The primary observer repeated the measurements on the same subset after a 2-week interval to assess intraobserver reliability.

Standardization of Planes: The oblique sagittal plane was reconstructed strictly parallel to the glenoid articular surface. The “Y-shaped” view, where the coracoid process base, scapular spine, and scapular body meet, was identified as the reference slice for all supraspinatus muscle measurements [14, 17, 18].

### Measurement of various factors based on CT

**Fig. 1:**
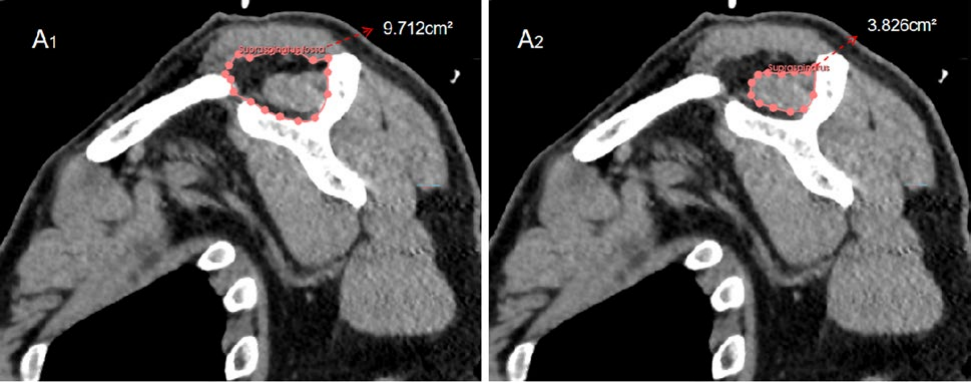
Measurement of supraspinatus fossa and the supraspinatus muscle. We measured the cross-sectional areas of the supraspinous fossa and the supraspinatus muscle in the outermost oblique sagittal plane through the boundary between muscle and bone. A1: The area of the supraspinous fossa was measured using 3D- slicer to be 9.712 cm². A2: The area of the supraspinatus muscle was 3.826 cm²

The supraspinatus occupation ratio(SSPOR) was calculated on the reference oblique sagittal slice as the ratio of the cross-sectional area of the supraspinatus muscle to the area of the supraspinatus fossa. This specific slice is defined where the scapular body intersects with the spine of the scapula on the oblique sagittal plane. The supraspinatus fossa is the nearly enclosed space bounded superiorly by the clavicle, scapula, and the deep surface of the trapezius muscle. The cross-sectional areas of both the supraspinatus muscle and the fossa at this level were manually outlined using software, which then automatically computed their areas to derive the ratio (Fig. 1). Supraspinatus fatty infiltration(SSPFI) was assessed on CT images according to the classification proposed by Goutallier et al. The Goutallier stages are defined as follows: Grade 0: The muscle exhibits completely normal, homogeneous soft-tissue density without any low-attenuation fatty streaks. Grade 1: Some minimal, linear or stippled low-attenuation (fatty) streaks are present within the muscle. Grade 2: There is evident fatty infiltration, but the amount of fat remains less than the amount of muscle tissue (i.e., fat < 50%). Grade 3: The quantity of fat is approximately equal to the quantity of muscle tissue (i.e., fat ≈ 50%). The muscle density is markedly heterogeneous, and muscle volume often begins to decrease (atrophy). Grade 4: The amount of fat substantially exceeds that of muscle tissue (i.e., fat > 50%). The muscle is severely atrophied and is largely replaced by extensive areas of low-attenuation fat, with only few residual strands of soft-tissue density.

**Fig. 2:**
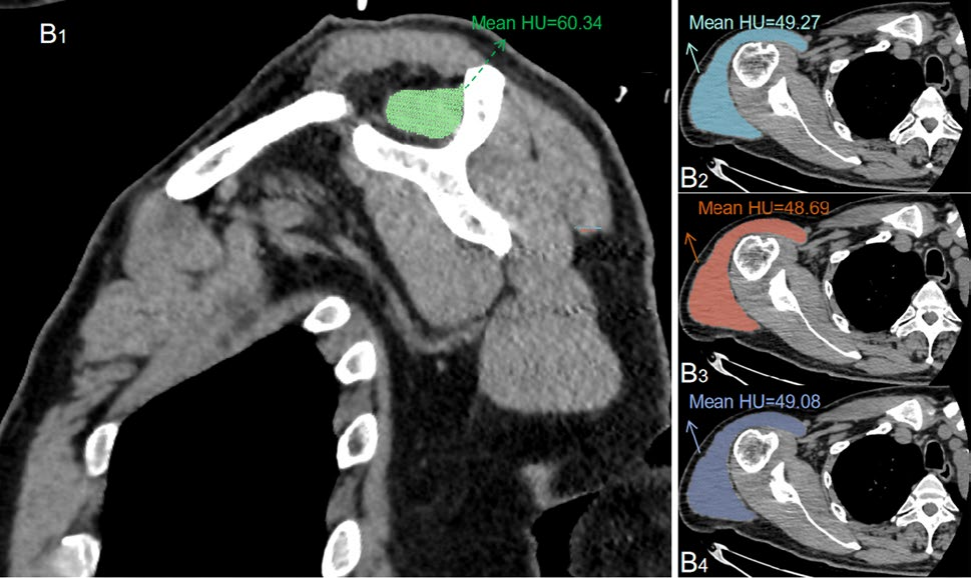
Measurement of HU values of Supraspinatus and deltoid .The Hounsfield Unit (HU) values of the supraspinatus muscle were measured on the most lateral oblique sagittal plane, and the HU values of the deltoid muscle were measured on the axial plane. B1: The entire supraspinatus muscle was delineated for HU measurement. The HU value of the supraspinatus muscle in this patient was 60.34. B2-B4: Based on three adjacent axial slices that fully visualized the deltoid muscle, its HU values were measured. The three HU measurement results were 49.27, 48.69, and 49.08, respectively

On the reference oblique sagittal slice, the mean Hounsfield Unit (HU) value within the outlined supraspinatus muscle region of interest (ROI) was recorded (SSPHU). On axial images, three consecutive slices at the mid-glenoid level showing a well-defined deltoid muscle contour were selected. The deltoid muscle was manually outlined on each slice, and the mean HU value was calculated (DHU)(Fig. 2). The ratio was then calculated as DHU / SSPHU [16]. The deltoid was measured on three slices to account for its morphological heterogeneity along the craniocaudal axis, whereas the supraspinatus was measured on a single, well-established, standardized slice for atrophy assessment [14, 17, 18].

**Fig. 3:**
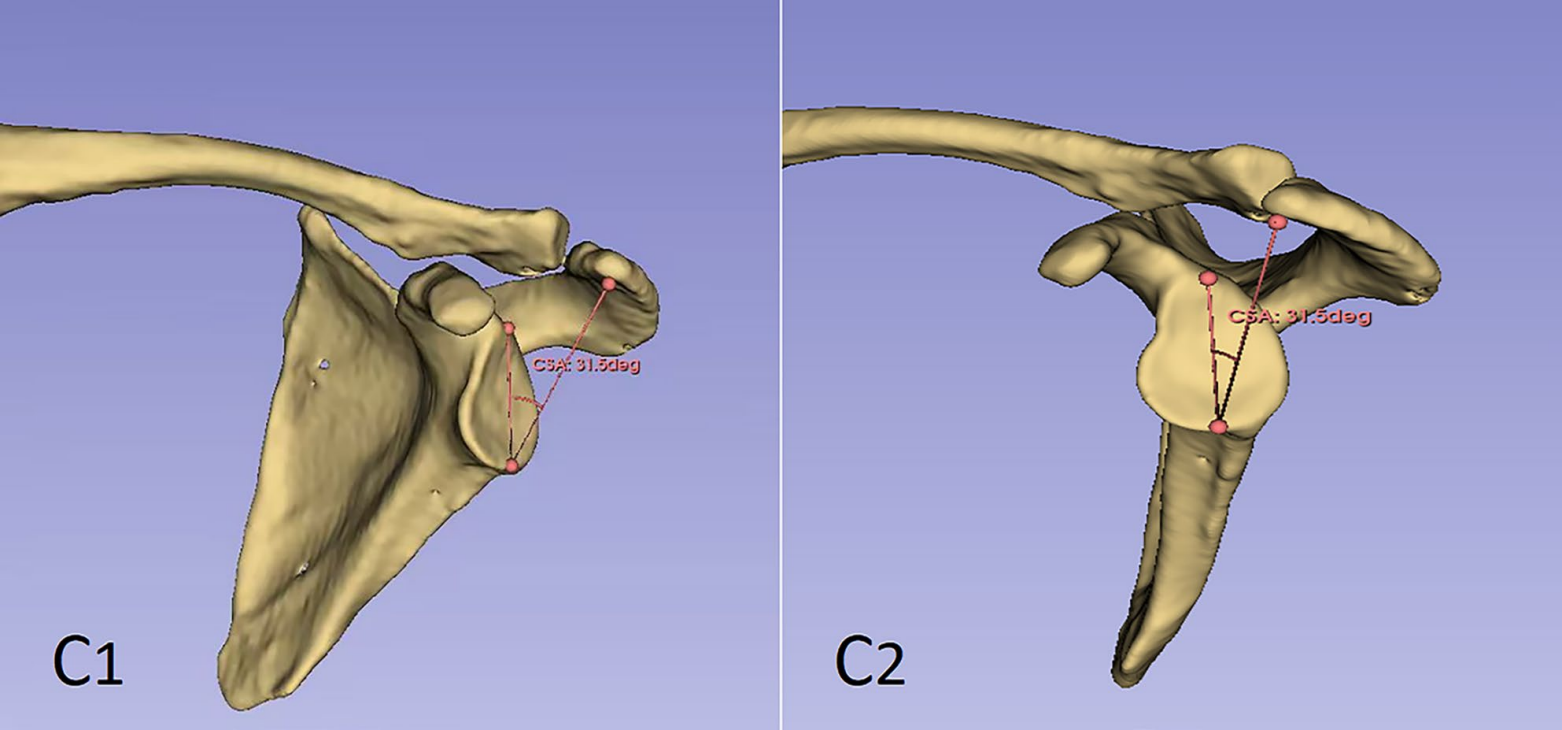
C1:Using 3D-slicer software, the scapula was reconstructed in 3D based on the patient's shoulder CT. Three points were placed at the upper and lower edges of the glenoid and the most lateral lower edge of the acromion, and the angle between the upper and lower edges of the glenoid and the line connecting the most lateral point of the acromion and the lower edge of the glenoid was measured. This angle was 31.5°.C2:Lateral view of the scapula

**Fig. 4:**
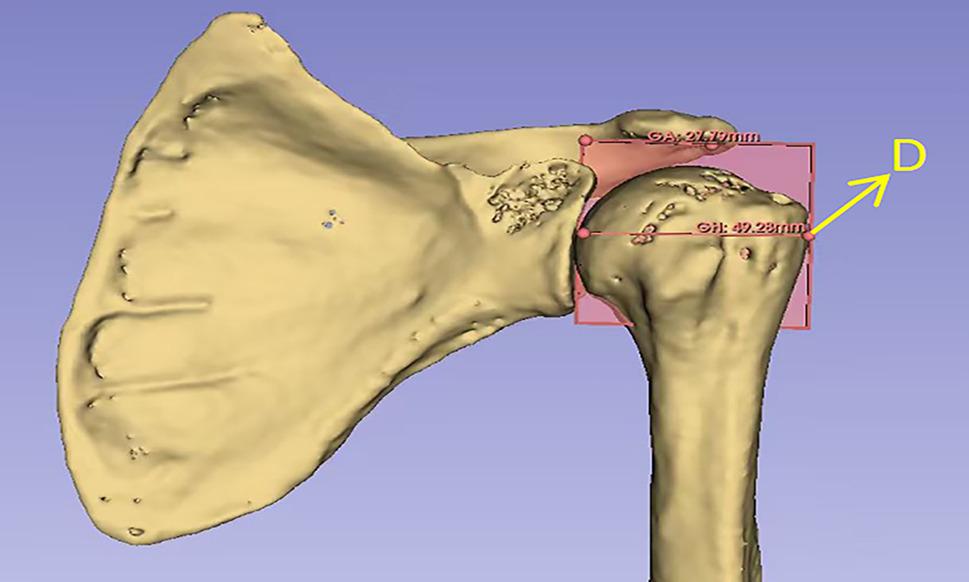
Using the 3D-Slicer software, the patient's scapula and humerus were reconstructed in 3D. A plane was established through two points at the upper and lower edges of the glenoid and the most lateral point of the acromion. The projection of the lateral-most point of the humerus onto this plane was designated as point D. The distance GH between point D and the upper and lower edges of the glenoid was measured as 49.28 mm, and the distance GA between the most lateral point of the acromion and the upper and lower edges of the glenoid was measured as 27.79 mm. The AI was calculated to be 0.56

The CSA was defined as the angle between the line connecting the superior and inferior margins of the glenoid and the line connecting the most lateral aspect of the acromion to the inferior glenoid margin(Fig. 3). The AI was defined as the ratio of the horizontal distance from the glenoid rim (a reference line drawn perpendicular to the plane of the glenoid) to the most lateral point of the acromion, to the horizontal distance from the same glenoid reference line to the most lateral point of the humeral head(Fig. 4).

### Observer reliability

For the HU ratio, interobserver reliability was excellent (ICC = 0.903), and intraobserver reliability was similarly excellent (ICC = 0.920).

### New scoring design

The scoring system for rotator cuff tears was developed utilizing patient clinical characteristics and various measurements derived from CT imaging. Receiver operating characteristic (ROC) curve analysis was employed to determine the optimal cut-off values for the predictive variables, with the Youden index used to define the threshold. Subsequently, a multivariable logistic regression model was applied to calculate the odds ratios (ORs) for a posterosuperior rotator cuff tear. Points were assigned to each predictor by rounding the natural logarithm of the OR to the nearest integer, a method that creates a clinically practical point score proportional to each variable’s contribution to the outcome risk. thereby establishing the RCT-PT score.

### Statistical analysis

All statistical analyses were performed using SPSS (version 20.0; IBM). A p-value of < 0.05 was considered statistically significant. In the univariate analysis, comparisons of means were conducted using the Student’s t-test. Continuous variables were analyzed using either the independent samples t-test or the Mann-Whitney U test, depending on their distribution. Categorical variables were compared using the Chi-square test or Fisher’s exact test, as appropriate. For the multivariate analysis, logistic regression was employed to identify independent variables associated with rotator cuff tears and to estimate their odds ratios (ORs). The ORs derived from the logistic regression model were used to determine the score assigned to each independent risk factor. The performance of this scoring system was then evaluated by applying it to the study cohort; its sensitivity and specificity were validated using values obtained from multiple imputation and ROC curve analysis, calculating the AUC, sensitivity, and specificity at the optimal cut-off.

## Results

**Fig. 5:**
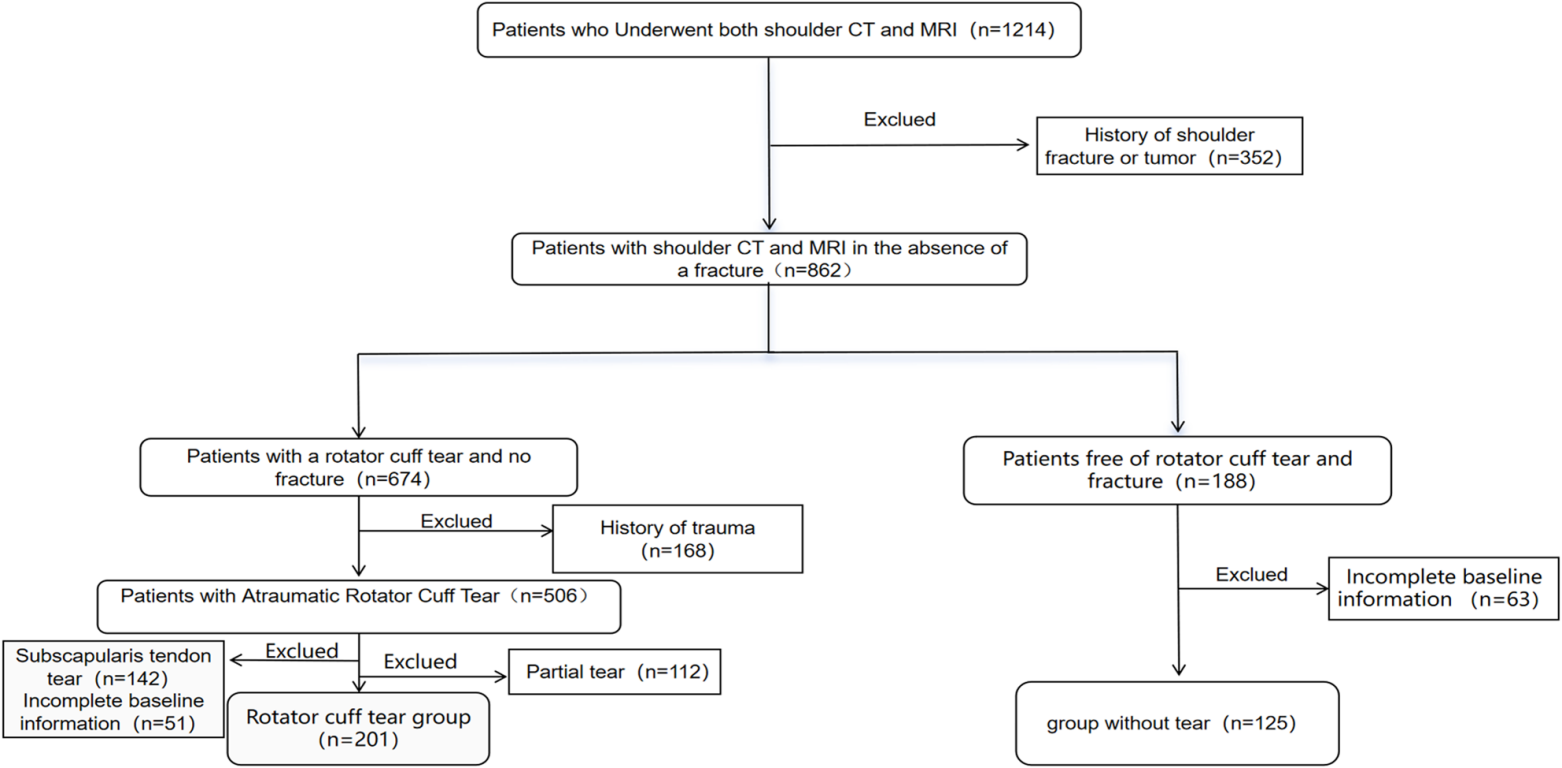
Flowchat. CT: Computer tomography; MRI: Magnetic resonance imaging

The final study cohort consisted of 201 patients in the rotator cuff tear group (mean age 53.7 ± 13.1 years; 130 female, 71 male) and 125 patients in the control group (mean age 43.3 ± 11.8 years; 57 female, 68 male). The two groups differed significantly in age and sex distribution(Fig. 5).

### Univariate analysis of each variable

Univariate analysis (Table 1) identified the following factors influencing rotator cuff tears: age (*p* < 0.01), sex (*p* < 0.01), supraspinatus occupation ratio (*p* < 0.01), critical shoulder angle (*p* < 0.01), deltoid-to-supraspinatus HU ratio (*p* < 0.01), acromial index (*p* < 0.01), symptom duration (*p* < 0.01), and Goutallier grade (*p* < 0.01). In contrast, body mass index (BMI, *p* = 0.59), diabetes (*p* = 0.104), hypertension (*p* = 0.737), and frozen shoulder (*p* = 0.66) were not significantly different. Furthermore, all physical examination findings—including the Hug-up test (*p* < 0.01), Drop arm sign (*p* < 0.01), Jobe test (*p* < 0.01), and External rotation lag test (*p* < 0.01) demonstrated statistically significant differences between the two groups.

**Table 1:** Comparison of clinical information between the two groups of patients

	No- RCT group	RCT group	T(Z)	*p*
Age, y	43.29 ± 11.805	53.65 ± 13.095	-10.979	< 0.01
Sex, n (%)	-	-	-	< 0.01
Male	68	71	-	-
Female	57	130	-	-
BMI, kg/m²	25.15 ± 2.94	24.84 ± 2.86	0.54	0.59
SSPOR (%), mean ± SD	76.03 ± 7.25	57.99 ± 12.99	-11.774	< 0.01
CSA °, mean ± SD	33.48 ± 2.856	37.36 ± 3.477	-9.573	< 0.01
DHU/ SSPHU, mean ± SD	0.83 ± 0.12	0.97 ± 0.28	-5.3	< 0.01
AI, mean ± SD	0.76 ± 0.086	0.51 ± 0.41	-7.327	< 0.01
DOS(m), mean ± SD	7.27 ± 13.90	12.55 ± 20.21	-4.423	< 0.01
SSPFI, n (%)	-	-	-	< 0.01
Stage 0	22(17.6)	1(0.5)	-	-
Stage 1	91(72.8)	50(24.9)	-	-
Stage 2	10(8)	116(57.7)	-	-
Stage 3	2(1.6)	29(8.6)	-	-
Stage 4	0(0)	5(2.5)	-	-
Diabetes, n (%)	9(7.2)	26(12.94)	-	0.104
Hypertension, n (%)	81(40.3)	48(38.4)	-	0.737
Frozen shoulder, n (%)	51(40.8)	87(43.28)	-	0.66
Hugup, n (%)	36(28.8)	181(90.05)	-	< 0.01
Dat, n (%)	27(21.6)	131(68.16)	-	< 0.01
Hawkins test, (%)	31(24.8)	119(59.20)	-	< 0.01
Jobe test, (%)	35(28.0)	158(78.61)	-	< 0.01
Er lag sign, (%)	26(20.8)	154(76.62)	-	< 0.01

*SSPOR* Supraspinatus Occupancy Rate, *CSA* Critical Shoulder Angle, *DHU/SSPHU* Deltoid Hounsfield Units /Supraspinatus Hounsfield Units, *DOS* Duration Of Symptoms, *AI* Acromion Index, *SSPFI* Supraspinatus Fatty Infiltration, *SD* Standard Deviation, *BMI* Body Mass Index, *Dat* Drop arm test, *Er lag sign* External rotation lag sign

### Independent risk factors

Multivariable logistic regression analysis (Table 2) identified the following independent risk factors: age, fatty infiltration grade (Goutallier grade), symptom duration, critical shoulder angle, and acromial index, with physical examinations including the Hug-up test, Drop arm sign, and External rotation lag test (Table 3) also identified as independent risk factors. Conversely, the supraspinatus occupation ratio was determined to be a protective factor. The deltoid-to-supraspinatus HU ratio was not significant in the multivariable model (*p* = 0.806).

**Table 2:** Risk factors associated with CT-based rotator cuff tears in multivariate analysis

variable	B	SE	Wald χ²	*p*	OR(Exp(B))	95% CI(OR)
Age	0.086	0.020	18.109	< 0.01	1.090	[1.047–1.134]
Sex	0.463	0.403	1.318	0.251	1.589	[0.721-3.500]
SSPFI	0.812	0.409	3.950	0.047	2.252	[1.011–5.016]
DOS	0.035	0.014	6.252	0.012	1.036	[1.008–1.065]
DHU/ SSPHU	-0.004	0.016	0.061	0.806	0.996	[0.965–1.028]
SSPOR	-0.128	0.028	20.982	< 0.01	0.880	[0.833–0.929]
CSA	0.162	0.073	4.833	0.028	1.175	[1.018–1.357]
AI	0.065	0.031	4.520	0.034	1.068	[1.005–1.134]
constant	-7.218	3.344	4.659	0.031	0.001	-

*SSPFI * Supraspinatus Fatty Infiltration, *DOS* Duration Of Symptoms, *DHU/SSPHU* Deltoid Hounsfield Units /Supraspinatus Hounsfield Units, *SSPOR* Supraspinatus Occupancy Rate, *CSA * Critical Shoulder Angle, *AI* Acromion Index, *OR* Odds Ratio

**Table 3:** Physical examinations associated with rotator cuff tears in multivariate analysis

variable	B	SE	Wald χ²	*p*	OR(Exp B )	95% CI(OR)
Hugup	2.403	0.429	31.388	< 0.01	11.081	[4.771–25.641]
Dat	1.139	0.536	4.515	0.036	3.124	[1.092–8.931]
Hawkins test	−0.899	0.539	2.786	0.095	0.407	[0.141–1.170]
Jobe test	−0.349	0.468	0.556	0.456	0.705	[0.282–1.766]
Er lag sign	1.517	0.397	14.591	< 0.01	4.558	[2.093–9.972]
constant	−1.738	0.286	42.118	< 0.01	0.176	-

*Dat * Drop arm test, *Er lag sign* External rotation lag sign

### The thresholds of various risk factors and the AUC

The study determined the following optimal cut-off values for the predictors: age (50 years), fatty infiltration grade (Goutallier Stage 2), symptom duration (4.5 months), supraspinatus occupation ratio (0.69), critical shoulder angle (34.5°), and acromial index (0.80) (Table 4). The AUCs for individual predictors ranged from 0.645 to 0.888. However, prediction based on any single factor is inherently limited. Therefore, a combined approach that integrates these risk factors is necessary for a more robust and accurate prediction model.

**Table 4:** Thresholds and AUC of each influencing factor

Risk factors	Region	Standard Error	Asymptotic Significance	95% Asymptotic CI		Threshold
				Lower	Upper	
Age	0.861	0.022	<0.01	0.818	0.905	50
SSPFI	0.848	0.022	<0.01	0.804	0.891	2
DOS	0.645	0.032	<0.01	0.583	0.707	4.5
SSPOR	0.888	0.017	<0.01	0.854	0.922	0.69
CSA	0.814	0.024	<0.01	0.767	0.861	34.5
AI	0.741	0.030	<0.01	0.683	0.799	0.80

SSPFI: Supraspinatus Fatty Infiltration; DOS: Duration Of Symptoms; SSPOR：Supraspinatus Occupancy Rate; CSA: Critical Shoulder Angle; AI: Acromion Index

### Scoring system design

The CT-based rotator cuff tear prediction (RCT-PT) score was determined by weighting each variable according to its odds ratio (OR) for influencing rotator cuff tear risk (Table 5). Points were assigned as follows: age ≥ 50 years (OR = 1.090, 1 point), fatty infiltration grade ≥Stage 2 (OR = 2.252, 2 points), symptom duration ≥ 4.5 months (OR = 1.036, 1 point), critical shoulder angle ≥ 34.5° (OR = 1.175, 1 point), and acromial index ≥ 0.80 (OR = 1.068, 1 point). The supraspinatus occupation ratio, identified as the strongest protective factor, was assigned 1 point when < 0.69 (OR = 0.880). Points based on physical examination findings were assigned as follows: positive Hug-up test (OR = 11.061, 3 points), positive Drop arm sign (OR = 3.124, 1 point), and positive External rotation lag test (OR = 4.558, 1 point) (Table 6).

**Table 5:** Weighted scores of CT-based influencing factors after multivariate analysis

	*P*	OR	95% CI	Points of each parameter
Age ≥ 50	< 0.01	1.090	[1.047–1.134]	1
SSPFI ≥ 2	0.047	2.252	[1.011–5.016]	2
DOS ≥ 4.5	0.012	1.036	[1.008–1.065]	1
SSPOR < 0.69	< 0.01	0.880	[0.833–0.929]	1
CSA ≥ 34.5°	0.028	1.175	[1.018–1.357]	1
AI ≥ 0.80	0.034	1.068	[1.005–1.134]	1

*SSPFI* Supraspinatus Fatty Infiltration, *DOS* Duration Of Symptoms, *SSPOR* Supraspinatus Occupancy Rate, *CSA* Critical Shoulder Angle, *AI* Acromion Index, *OR* Odds Ratio

**Table 6:** Weighted scores of physical examinations after multivariate analysis

	*P*	OR	95% CI	Points of each parameter
Hugup	< 0.01	11.061	[4.771–25.641]	3
Dat	0.036	3.124	[1.092–8.931]	1
El lag test	< 0.01	4.558	[2.093–9.972]	1

*Dat* Drop arm test, *Er lag sign * External rotation lag sign

### Research population under the RCT-PT Scoring system

When the combined score from CT-based/clinical factors and physical examination findings was considered as the total score and applied to the study population, the scoring system (RCT-PT) showed that the rotator cuff tear group had a mean score of 9.56 (range: 1–12), while the control group (non-tear group) had a mean score of 2.96 (range: 0–11) (Table 7). Using a cut-off score of 6.5, the system demonstrated a sensitivity of 86.1% and a specificity of 86.4% in predicting rotator cuff tears.

**Table 7:** Implementation of the New Scoring System in the Study Cohort

Total Score^a^	No- RCT group(*n*^b^)	RCT group(*n*^b^)	Sensitivity,%	Specificity,%	ppv,%
0	20	0	100.0	0.0	61.7
1	37	2	100.0	16.0	65.7
2	21	6	99.0	45.6	74.5
3	5	6	96.0	62.4	80.4
4	8	5	93.0	66.4	81.7
5	7	3	90.6	72.8	84.3
6	10	6	89.1	78.4	86.9
7	4	13	86.1	86.4	91.1
8	3	10	79.6	89.6	92.5
9	5	16	74.6	92.0	93.8
10	3	25	66.7	96.0	96.4
11	2	55	54.2	98.4	98.2
12	0	54	26.9	100.0	100.0

*Ppv* positive predictive value

a: CT factor score + physical examination score

b: Distribution of patients across the new scoring system

### THE RCT-PT is used to study the results obtained from the population

**Fig. 6:**
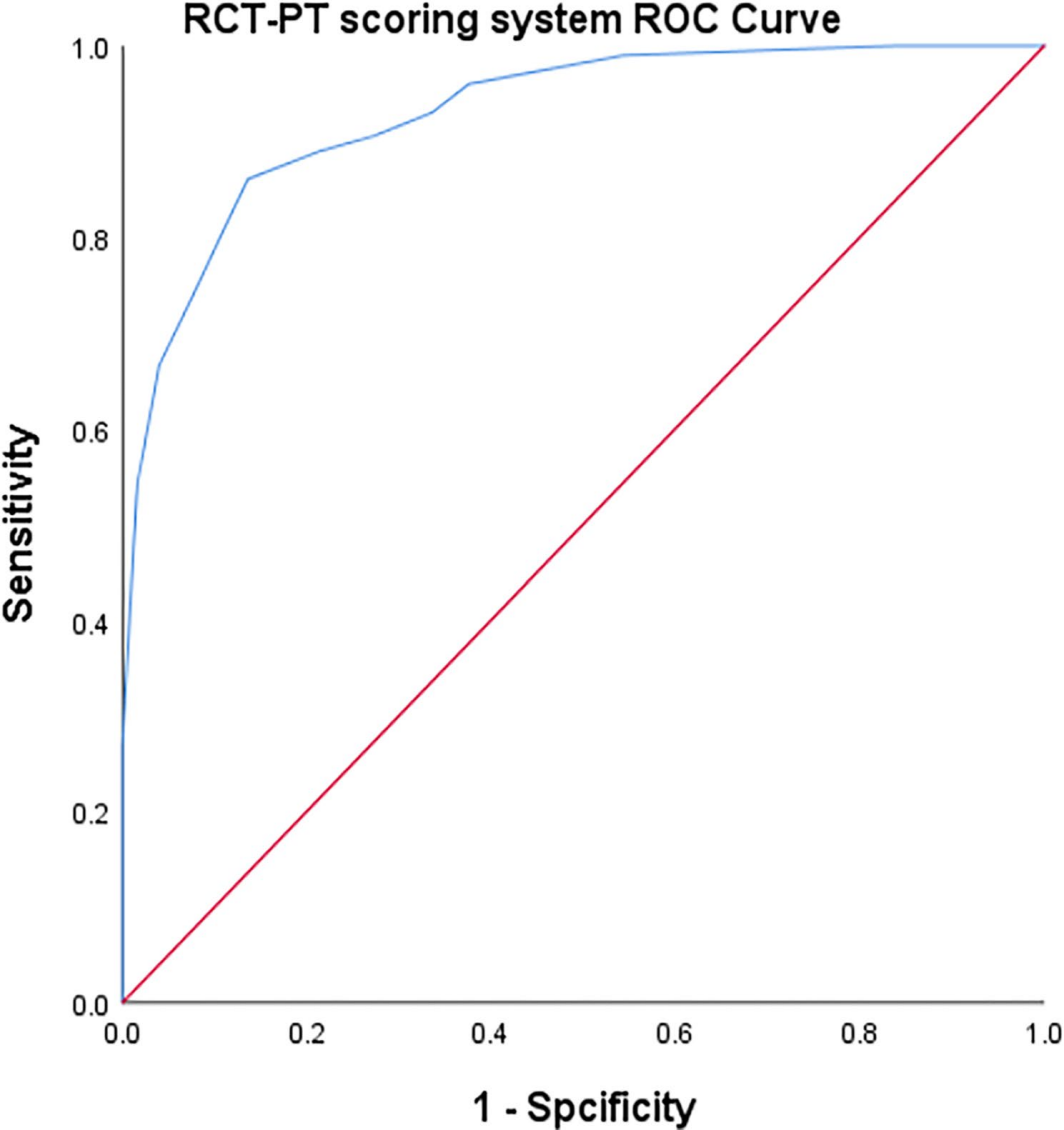
The receiver operating characteristic (ROC) analysis in the study population for the new scoring system (RCT-PT) resulted in an area under the curve (AUC) of 0.930 (P < .001), with a determined cutoff value 6.5, achieving a sensitivity of 86.1% and specificity of 86.4%

In the study population, the Receiver Operating Characteristic (ROC) analysis of the RCT-PT scoring system yielded an area under the curve (AUC) of 0.930 (*p* < 0.01) (Table 8). The determined cut-off value was 6.5, which corresponded to a sensitivity of 86.1% and a specificity of 86.4% (Fig. 6).

**Table 8:** Analysis of ROC Curve Results for Predicting RCT with RCT-PT

	Cutoff Value	Youden Index J	Sensitivity	Specificity	AUC
Scoring	≥ 6.5	0.725	0.861	0.864	0.930

*AUC * area under the curve

## Discussion

This study developed and internally validated a novel scoring system (RCT-PT) that integrates patient characteristics, physical examination findings, and standard CT-derived morphological parameters to predict the likelihood of a full-thickness degenerative posterosuperior rotator cuff tear. The system demonstrated excellent diagnostic performance (AUC 0.93), with a score of ≥ 6.5 providing high sensitivity and specificity. Our findings confirm the independent predictive value of previously established factors such as CSA, AI, Goutallier grade, and age [4, 5, 19, 20], while incorporating them into a single, clinically actionable tool. The primary finding of this study was the identification of risk factors associated with degenerative posterosuperior rotator cuff tears, including age, Goutallier grade of fatty infiltration in the supraspinatus muscle, symptom duration, critical shoulder angle, and acromial index. These independent risk factors were used to develop a CT- and clinically-based score (range 0–7 points), while physical examination findings were assigned a separate score (range 0–5 points). Together, these components constitute the RCT-PT—an auxiliary diagnostic scoring system with a total range of 0 to 12 points. At the optimal cut-off value of 6.5 points, this scoring system demonstrated a positive predictive value of 91.1% and a sensitivity of 86.1% in diagnosing rotator cuff tears.

The clinical utility of this score lies in its ability to risk-stratify patients. A score of ≤ 3 suggests a low probability of a significant tear, supporting initial conservative management. A score between 4 and 6 indicates a moderate risk, warranting closer clinical monitoring and patient education. A score of ≥ 7, however, raises a high suspicion for a full-thickness posterosuperior RCT. In a patient with glenohumeral arthritis being evaluated for shoulder arthroplasty, such a score from a standard pre-operative planning CT could significantly influence surgical strategy. A high suspicion of an irreparable RCT might steer the surgeon towards a reverse total shoulder arthroplasty rather than an anatomic arthroplasty, a critical decision that typically relies on MRI [21, 22]. This represents the most valuable niche for our tool: providing actionable soft-tissue insight from a bony-imaging study.

Despite existing literature on risk factors like age, BMI, acromial index, critical shoulder angle, and diabetes [4, 5, 19, 20], a comprehensive scoring system that integrates these variables based on shoulder CT to predict rotator cuff tears is still lacking. Prior studies have identified elevated acromial index and critical shoulder angle as significant risk factors for rotator cuff tears. It has been documented that the mean CSA is approximately 36.7° in patients with full-thickness tears, 34.6° in those with partial-thickness tears, and 33.1° in individuals without rotator cuff tears [4, 5]. Our identified CSA cut-off of 34.5° is consistent with these values reported in the literature [6]. Similarly, our AI cut-off of 0.80 mirrors the threshold associated with rotator cuff pathology [5, 23]. Armstrong first proposed that mechanical conflict between the acromion and the supraspinatus tendon was the cause of supraspinatus tendon degeneration; this concept was subsequently popularized by Neer [24, 25]. The critical shoulder angle (CSA) represents the extent of acromial coverage over the humeral head. Consequently, a higher CSA value increases the superiorly directed vector of the deltoid muscle, leading to elevated stress on the rotator cuff [26]. This functional effect is considered more relevant than direct contact between the acromion and the rotator cuff. Based on MRI and radiological assessments respectively, Nyffeler et al. and Banas et al. reported on the lateral acromial angle and the acromial index. Their studies established a statistically significant relationship where a smaller lateral acromial angle and a larger acromial index were associated with a higher incidence of shoulder impingement syndrome and subacromial pathology [27, 28].

Since the introduction of the grading system by Goutallier in 1994, fatty infiltration and muscle atrophy have been consistently linked to postoperative outcomes following arthroscopic surgery [12, 29–31]. However, no studies have attempted to utilize fatty infiltration and muscle atrophy for predicting rotator cuff tears. The strong predictive weight of the Goutallier grade (2 points) and occupation ratio (1 point as a protective factor) reinforces the central role of muscle quality in the pathophysiology of chronic tears [12, 14]. Stengaard et al. and Frich et al. demonstrated that the supraspinatus muscle exhibits early acute inflammation, initial muscle degeneration, and significant signs of fatty infiltration following a rotator cuff tear [32, 33]. Furthermore, Wang et al. showed that delayed rotator cuff repair leads to persistent muscle atrophy and fatty infiltration, particularly when repair is delayed beyond 6 weeks, resulting in poorer shoulder function [34]. Concurrently, Rubino et al. confirmed that significant muscle atrophy and fatty infiltration are evident as early as six weeks after supraspinatus tendon detachment. Moreover, the observed muscle atrophy and fatty infiltration in rotator cuff tears progress from the tendon-muscle junction towards the muscle origin over time [35]. While our cross-sectional data cannot demonstrate this temporal progression directly, our finding of significantly higher grades and lower occupation ratios in the tear group is fully consistent with this established concept. The present study found that fatty infiltration in the control group (without rotator cuff tears) was primarily distributed at Goutallier grades 0 and 1. In contrast, the tear group showed a significant shift towards higher grades (grade 2 and 3), indicating that degenerative changes and minor fatty infiltration can occur even in intact rotator cuffs. The mean supraspinatus occupation ratio was approximately 76% in the control group compared to about 58% in the tear group. This finding further confirms that muscle atrophy progresses from the tendon-muscle junction towards the muscle origin and advances continuously with the presence and progression of a rotator cuff tear.

Previous studies have reported associations between rotator cuff pathology and factors such as age, hypertension, and diabetes [36–38]. Consistent with this literature, our study identified age as one of the strongest risk factors, with rotator cuff tear risk steadily increasing with advancing age, reflecting the combined effects of biological degeneration and cumulative mechanical stress on tendons over time. However, contrary to some previous reports, we found no statistically significant differences in the prevalence of hypertension, diabetes, or frozen shoulder between the two patient groups. This lack of association, while seemingly contrary to some meta-analyses, may reflect insufficient statistical power in our specific cohort or confounding by the strong age effect. The relationship between these comorbidities and rotator cuff tears, including the underlying mechanisms, may require further investigation in future studies.

Ashry et al. found that in intact rotator cuffs, muscle atrophy and fatty infiltration increase with age. However, in the presence of a tear, atrophy disproportionately affects the supraspinatus compared to the deltoid. Consequently, using the deltoid as an internal reference for comparative assessment may be more reliable than evaluating the supraspinatus in isolation [16, 39]. We initially hypothesized that the deltoid-to-supraspinatus HU ratio would serve as a sensitive marker, accounting for individual variation in systemic muscle health. In our study, while the deltoid-to-supraspinatus Hounsfield unit (HU) ratio showed a significant difference between the two patient groups on univariate analysis, it was not identified as an independent predictor in the multivariable logistic regression model (*p* = 0.806). This is likely due to multicollinearity with the Goutallier grade and occupation ratio, which are more direct measures of the same pathological processes within the supraspinatus. The HU ratio may capture redundant information once these direct measures are included. A plausible explanation for this finding is that the processes of fatty infiltration and muscle atrophy concurrently alter the HU values of both the deltoid and supraspinatus muscles. This likely induced a high degree of multicollinearity between the HU ratio and the other direct measures of atrophy and infiltration (e.g., Goutallier grade, occupation ratio). Consequently, the HU ratio was unable to demonstrate a statistically independent contribution within the multivariable model.

This study has several limitations. First, it was a single-center investigation with a relatively small sample size that lacked an a priori power analysis. The sample, while adequate for model development, may not be representative of the general population. Patient clinical information did not include potential influencing factors such as hypercholesterolemia, smoking, or alcohol consumption. Second, the retrospective case-control design and reliance on historical records make the study susceptible to selection and information bias. The significant age difference between groups, while adjusted for in multivariable analysis, raises the possibility of residual confounding. The selection of cases and controls may not fully represent the general population, and data collection—including radiographic measurements and history taking—was not performed in a blinded manner, potentially introducing observer bias. Third, the assessment of “symptom duration” was based on patient recall, which is subject to recall bias. Furthermore, rotator cuff tears were treated as a binary outcome (present/absent) without considering tear size, location, or morphology, factors that might influence the weight of risk factors. Fourth, our study did not include a comparison with ultrasound, which is a highly accurate, cost-effective diagnostic tool for full-thickness RCTs [40]. The scoring system is not intended to compete with ultrasound but to fill a specific gap when only CT is available. Finally, it must be emphasized that this risk scoring system is intended as a screening and risk stratification tool, not a definitive diagnostic method. A high score should be regarded as an indication for more precise examination (e.g., MRI) and cannot replace the clinical gold standard. Furthermore, the scoring system was developed and tested on the same cohort. External validation in a separate, prospective, multicenter cohort is essential before it can be recommended for widespread clinical use.

In conclusion, while recognizing the clinical value of this study, its limitations should be acknowledged. Future research should focus on validating and refining this scoring system through multicenter, large-scale, prospective cohort studies. Additionally, incorporating more comprehensive clinical variables, utilizing artificial intelligence for automated and precise measurement of imaging parameters, and exploring integration with molecular biomarkers represent promising directions for developing next-generation predictive models for rotator cuff tears.

## Conclusion

We have developed a practical 12-point scoring system (RCT-PT) based on routine shoulder CT and clinical factors that effectively stratifies the risk of a full-thickness degenerative posterosuperior rotator cuff tear. The score demonstrates excellent predictive accuracy within our cohort. In patients with contraindications to MRI or for whom CT is the only available advanced imaging—particularly those being evaluated for shoulder arthroplasty—this score can provide critical decision-support, aiding in surgical planning and the decision for further, more definitive diagnostic evaluation. External validation is required prior to broad clinical implementation. A numerical scoring system based on shoulder CT and clinical factors was developed to predict rotator cuff tears. For clinical application, a patient score of ≤ 3 suggests a low probability of a rotator cuff tear, recommending initial observation or conservative management; a score between 4 and 6 indicates a moderate risk of tear, suggesting guided self‑monitoring (e.g., for weakness or night pain) and short‑term follow‑up; when the score reaches ≥ 7, there is a high suspicion of a posterosuperior rotator cuff tear, warranting prompt further evaluation with shoulder MRI or arthrography along with specialist assessment. Furthermore, this system provides a diagnostic alternative and reliable risk assessment for patients who are unable to undergo MRI due to specific contraindications.

## Data Availability

Data is available via a request to the corresponding author.

## Abbreviations

RCT: Rotator cuff tear
MRI: Magnetic resonance imaging
CT: Computed tomography
BMI: Body mass index
CSA: Critical shoulder angle
AI: Acromion index
HU: Hounsfield Unit
DOS: Duration of symptoms
AUC: Area under the curve
OR: Odds ratio
AUC: Area under curve
RCT-PT: Posterosuperior rotator cuff tear
US: Ultrasound
SSPOR: Supraspinatus occupation ratio
SSPFI: Supraspinatus fatty infiltration
ROI: Region of Interest
SSPHU: Supraspinatus Hounsfield Unit
DHU: Deltoid Hounsfield Unit
DHU / SSPHU: Deltoid / Supraspinatus HU
ICC: Intraclass correlation coefficient
ROC: Receiver Operating Characteristic
